# Neck circumference in relation to glycemic parameters: a systematic review and meta-analysis of observational studies

**DOI:** 10.1186/s13098-019-0445-7

**Published:** 2019-06-25

**Authors:** Parvane Saneei, Farnaz Shahdadian, Sajjad Moradi, Abed Ghavami, Hamed Mohammadi, Mohammad Hossein Rouhani

**Affiliations:** 1 Department of Community Nutrition, School of Nutrition and Food Science, Food Security Research Center, Isfahan University of Medical Sciences, Isfahan, Iran; 2 Students’ Research Committee, Isfahan University of Medical Sciences, Isfahan, Iran; 3 Department of Clinical Nutrition, School of Nutrition and Food Science, Food Security Research Center, Isfahan University of Medical Sciences, Isfahan, Iran; 4 Halal Research Center of IRI, FDA, Tehran, Iran; 5 Nutritional Sciences Department, School of Nutritional Sciences and Food Technology, Kermanshah University of Medical Sciences, Kermanshah, Iran

**Keywords:** Neck circumference, Fasting plasma glucose, Insulin levels, Insulin resistance, Glycated hemoglobin

## Abstract

**Background:**

Recent studies have suggested that neck circumference (NC) is a supplemental screening measure for diagnosing metabolic complications and might be associated with glycemic parameters. The aim of the present study was to to evaluate the association between NC and glycemic parameters.

**Methods:**

We systematically searched the electronic databases (including MEDLINE, Scopus, EMBASE, and Google scholar) up to April 2018. Observational studies that reported correlation coefficient between NC and glycemic parameters were included in the analysis. A random effects model was used to estimate overall Fisher’s Z and 95% confidence interval of glycemic parameters including fasting plasma glucose (FBG), serum fasting insulin level, homeostasis model assessment-estimated insulin resistance (HOMA-IR) and glycated hemoglobin (HbA1c).

**Results:**

A total of 21 studies (44,031 participants) were eligible for including in the systematic review and meta-analysis. Significant correlations were found between NC and FBG (Fisher’s Z = 0.18; 95% CI 0.16, 0.21), serum fasting insulin level (Fisher’s Z = 0.34; 95% CI 0.26, 0.41), HOMA-IR (Fisher’s Z = 0.36; 95% CI 0.29, 0.43) and HbA1c (Fisher’s Z = 0.14; 95% CI 0.09, 0.20). Meta-regression analysis showed that NC were marginally associated with FBG in a linear manner (β = 0.008, P = 0.09); but not related to serum fasting insulin level, HOMA-IR, and HbA1c.

**Conclusions:**

This meta-analysis of cross-sectional studies showed that NC was positively correlated with glycemic parameters including FBG, serum fasting insulin level, HOMA-IR, and HbA1c. Further investigations with prospective design are required to confirm these findings.

## Background

Diabetes mellitus is a complex metabolic disease characterized by high serum glucose concentration and insulin resistance in target tissues and/or defects in insulin secretion [1]. It may lead to chronic complications such as nephropathy, retinopathy and neuropathy [2]. During the past decades, global prevalence of diabetes continues to rise in parallel with the rates of obesity [3]. Early detection of diabetes by appropriate screening methods may help to delay the vascular complications, especially in individuals who are at high risk for diabetes [2].

Obesity may lead to insulin resistance and development of type 2 diabetes [3]. Upper-body subcutaneous adipose tissue may confer additional risk for metabolic disorders beyond overall and abdominal obesity [4]. Neck circumference (NC) is a surrogate marker of upper-body subcutaneous fat distribution and closely correlated with various metabolic risk factors [5]. The associations between NC and components of the insulin resistance and metabolic syndrome have been investigated [6]. Therefore, NC may play a remarkable role in prediction of type 2 diabetes [7].

In the past decades, accumulating evidence showed that NC was independently associated with glycemic parameters, including fasting blood glucose (FBG), insulin levels, insulin resistance, and glycated hemoglobin (HbA1c). However, the results were inconsistent. In the Framingham Heart Study, Lee et al. found that NC was positively associated with fasting plasma glucose [8]. Likewise, NC was also related to glycemic parameters in a Chines elderly population [9], Japanese postmenopausal women [10], Chinese adults [11] and other populations [12]. However, some studies reported that NC was not significantly associated with fasting plasma glucose, insulin or insulin resistance [13–15]. So, it is not clear whether measurement of NC is a better predictor of type 2 diabetes compared with traditional adiposity measure. A recently published meta-analysis has evaluated the association between NC and risk of metabolic syndrome [16]. Although a positive association between NC and FBG—as a component of metabolic syndrome—was reported in this meta-analysis, several relevant studies have been missed in the search process [11, 12, 17]. Also, there was no summarizing report for the association between NC and other glycemic parameters. Therefore, we aimed to conduct a systematic review and meta-analysis to evaluate the correlation between NC and glycemic parameters including FBG, serum fasting insulin, homeostasis model assessment-estimated insulin resistance (HOMA-IR) and HbA1c.

## Methods

### Search strategy

We adhered to the meta-analysis of observational studies in epidemiology (MOOSE) guidelines in this systematic review and meta-analysis [18]. A comprehensive systematic literature search using the MEDLINE (Pubmed) (https://www.ncbi.nlm.nih.gov/pubmed), SCOPUS (https://www.scopus.com), EMBASE (https://www.elsevier.com) and Google scholar (https://scholar.google.com) databases was conducted up to April 2018 covering all published research providing evidence on the association between NC measurement and glycemic indices, including fasting plasma glucose, insulin levels, insulin resistance (or HOMA-IR) and HbA1c. Following terms were used: (“Neck Circumference”[Title/Abstract] AND (“Blood Glucose”[MeSh] OR “Blood Glucose”[Title/Abstract] OR “Fasting Plasma Glucose”[Title/Abstract] OR FBG[Title/Abstract] OR Insulin [MeSh] OR “Insulin Resistance”[MeSh] OR “Insulin Resistance”[Title/Abstract] OR “Insulin Levels”[Title/Abstract] OR HOMA-IR[Title/Abstract] OR “Glycated Hemoglobin A”[MESH] OR “Glycated Hemoglobin A”[Title/Abstract] OR HbA1C[Title/Abstract] OR “Diabetes Mellitus”[MeSh] OR “Diabetes Mellitus”[Title/Abstract] OR “Metabolic Syndrome”[MESH] OR “Metabolic Syndrome”[Title/Abstract]). After removing duplicates, two investigators (S.M. and P.S) independently conducted title and abstract screening and identified potentially relevant articles for the full-text review. No time or language restrictions were applied. In addition, a manual review of reference list of retrieved articles was carried out to identify additional relevant studies. Efforts were made to obtain additional data by contacting the authors.

### Inclusion criteria

Studies were included in the meta-analysis if they: [1] were conducted on adult (> 18 years) participants; [2] had a cohort or a cross-sectional design; [3] used NC as the exposure; and [4] reported Pearson or Spearman correlation coefficients between NC and FBG, insulin levels, HOMA-IR or HbA1C. If a same dataset had been analyzed in more than one publication, only the paper with the largest number of participants was included.

### Excluded studies

**Fig. 1:**
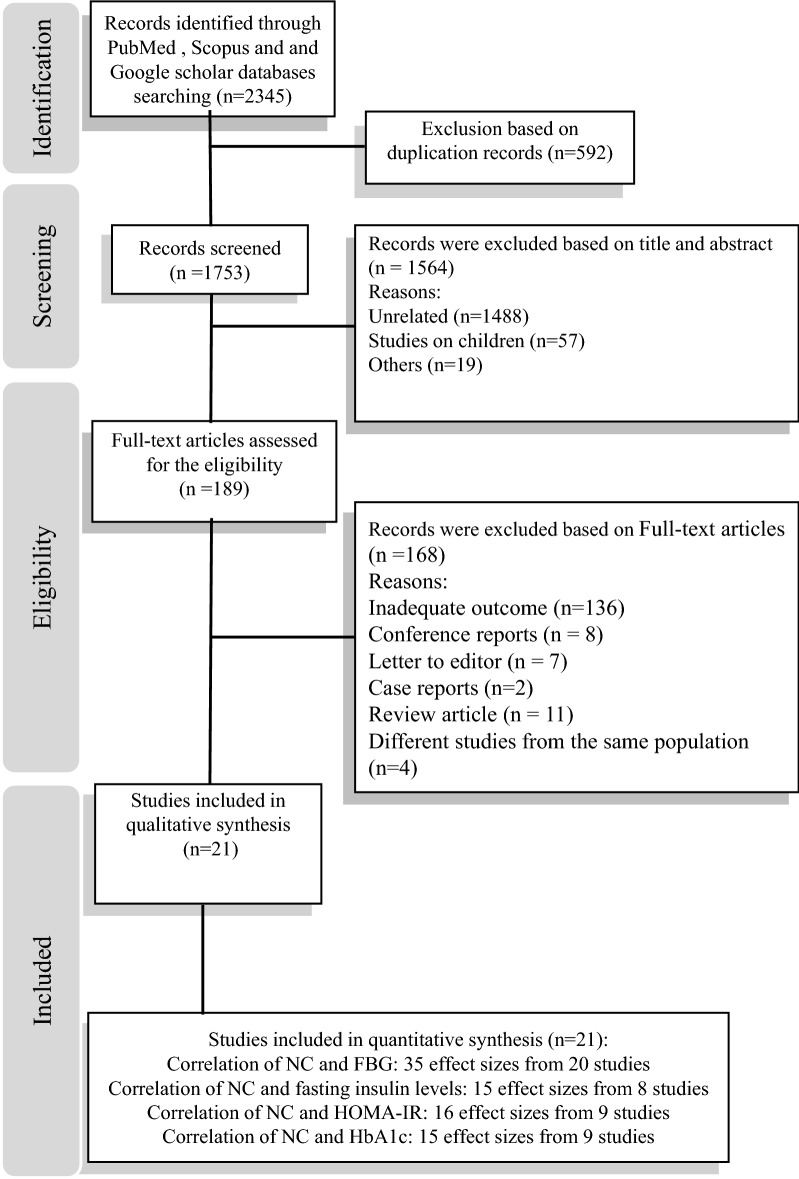
The flow diagram of study selection

We did not include gray literature including thesis, conference abstracts, qualitative, case-report and review studies. Researches on children and adolescents as well as studies that did not report correlation coefficient were also excluded. The flow diagram of study selection process is indicated in Fig. 1. Among eligible studies, Ben-Noun et al. had 2 reports in 2003 and 2006 from a same population [19, 20]. Therefore, study which enrolled larger sample size was included in the current analysis. Although the study population of 2 reports by Liang in 2014 and 2015 were also same [11, 21], values for correlation coefficient were not provided in 2014. Therefore, this report was excluded. Furthermore, 2 studies have been conducted by Aoi et al. in 2014 and 2016. In 2016, Aoi et al. reported the follow up results of the study that conducted in 2014 after 3 years [10, 22]. The baseline values for HbA1c and HOMA-IR reported by Aoi et al. in 2014 were included in the current analysis. Two reports from Framingham Heart Study cohort by Lee et al. and Preis et al. had a same population [8, 13]. In this case, the study by Lee et al. that had larger sample size was included in the present analysis.

### Data extraction

Following data were extracted from each study: the first author’s last name, publication year; study population, study name, location, gender, number of participants, age of participants, race or ethnicity, mean neck circumference and its standard deviation of participants, sampling method, statistical test used, assessment of outcomes, most fully adjusted Pearson or Spearman correlation coefficient between NC and each outcome and statistical adjustment for the potential confounding factors. Study selection and data extraction were conducted independently by two investigators (P.S. and S.M.).

### Quality assessment

We assessed study quality using the Newcastle–Ottawa quality assessment scale (adapted for cross sectional studies) [23]. This system allowed a total score of up to 10 points as the highest quality. Scores were derived through three aspects of each study including five scores for selection (representativeness of the sample, sample size, non-respondents and ascertainment of the exposure), two scores for comparability (considering confounding factors in study design or analysis) and three scores for outcome (assessment of the outcome and statistical test) in seven questions. Studies with scores above the median were classified as the high quality studies.

### Statistical methods

To perform the meta-analysis, we used correlation coefficients (reported for the relationship between NC and outcomes of interest) and sample sizes to calculate Fisher’s Z and its Standard Error of mean (SEM). Overall effect was derived from the method of DerSimonian and Laird [24] by using random effects model, which takes between-study variation into account. To find possible sources of heterogeneity, we conducted meta-regression based on mean NC as an effect modifier. Subgroup analysis based on gender, study location, sampling method, health status of participants, type of correlation coefficient and making adjustment was also done to find possible sources of heterogeneity. Between-subgroup heterogeneity was assessed by a fixed effect model. Statistical heterogeneity between studies was evaluated with Cochran’s Q test and I square (*I*^2^). Sensitivity analysis was used to explore the extent to which inferences might depend on a particular study. Publication bias was evaluated by Begg’s funnel plots. Formal statistical assessment of funnel plot asymmetry was done by Egger’s regression asymmetry test and Begg’s adjusted rank correlation test. Statistical analyses were conducted by using Stata version 11.2 (Stata Corp, College Station, TX). P values less than 0.05 were considered statistically significant.

## Results

### Study characteristics

Of 2345 articles identified by the initial search, 21 studies were eligible for including in the current systematic review and meta-analysis. Table 1 summarized the characteristics and quality score of included studies. Papers were published between 2002 and 2017. All studies had a cross-sectional design except for one case–control study. The total number of participants in these studies was 44,031 (19,710 males and 24,321 females) aged from 18 to 65 years. Mean NC were ranged from 31 to 44 cm. Six studies were conducted in the United States [12, 13, 17, 25–27], 10 in Asian countries [9, 11, 14, 22, 28–33], 2 in Middle-eastern countries [5, 19], 2 in Latin American societies [15, 34], and 1 in a European country [35]. Two investigations were conducted on women; one in men and others in both genders. The participants of 17 studies were healthy population and 3 investigations enrolled overweight or obese, severely obese individuals or clinically patients. One case–control study was conducted on both healthy and human immunodeficiency virus infected populations. Regarding sampling method, 4 studies used a consecutive method, 10 studies used random sampling techniques and 7 studies used a non-random method. In 10 studies, data were reported as age-adjusted. Two studies made further adjustments for gender, smoking status, physical activity, disease status and sex; while nine investigations did not make any adjustment for potential confounders. In case of quality of studies, the score quality of 7 studies was 8 and 12 studies were 9. The quality of 2 remained studies was a maximum of 10.

**Table 1:** Description of the studies included in the meta-analysis

First author (year)	Study/country	Subject and gender	Age range Or mean ± SD (y)	Race/ethnicity	Mean NC ± SD	Sampling method	Statistical test used	Reported or extracted data	Method of outcome assessment	Adjusted variables	Participants	Quality Score^a^
Dixon 2002 [25]	–/USA	F: 107	19–48	NR	40.6 ± 2.81	Consecutive	Pearson	FBG: 0.095 Insulin: 0.504 HbA1c: 0.095		Unadjusted	Severely obese premenopausal women	8/10
Ben-Noun 2003 [20]	–/Israel	M: 231 F: 330	≥18	Jewish	38.2 ± 2.7 34.2 ± 2.5	Consecutive	Pearson	FBG: 0.21 FBG: 0.44		Unadjusted	Healthy	8/10
Onat 2009 [5]	–/Turkey	M: 934	55.1 ± 12	NR	38.8 ± 2.9	Random	Pearson	FBG: 0.05 Insulin: 0.37 HOMA-IR: 0.35		Age	Healthy	9/10
		F: 978			34.8 ± 2.75			FBG: 0.11 Insulin: 0.27 HOMA-IR: 0.30				
Preis 2010 [13]	Framingham Heart Study cohorts/USA	M: 1720	28–62	NR	40.5 ± 2.9	Random	Pearson	FBG: 0.25 Insulin: 0.48 HOMA-IR: 0.49		Age	Healthy	9/10
		F: 1587			34.2 ± 2.8			FBG: 0.34 Insulin: 0.47 HOMA-IR: 0.51				
Fitch 2011 [26]	Massachusetts, USA	M: 43/F: 131	18–65	NR	37.0 ± 3.96	Non-random	Pearson	FBG: 0.27 Insulin: 0.18 HbA1c: 0.16		Unadjusted	HIV-infected	8/10
		M: 26/F: 128			36.4 ± 3.72			FBG: 0.27 Insulin: 0.26 HbA1c: 0.28			Healthy	
Zhou 2013 [28]	–/China	M: 2508 men	20–85	Asian	37.4 ± 2.46	Random	Pearson	FBG:0.177		Age	Healthy	9/10
		F: 1693			32.4 ± 2.24			FBG: 0.180				
Stabe 2013 [34]	BRAMS/Brazil	M: 301	18–60	Spanish	39.7 ± 2.9	Non-random	Spearman	FBG: 0.17 Insulin: 0.21 HOMA-IR: 0.30 HbA1c: 0.21		Age	Healthy	9/10
		F: 752			35.9 ± 2.8			FBG: 0.15 Insulin: 0.30 HOMA-IR: 0.42 HbA1c: 0.20				
Pokharel 2014 [17]	–/USA	M: 845	45–63	NR	43.1 ± 9.65	Non-random	Spearman	FBG: 0.15		Unadjusted	Retried NFL players/healthy	9/10
Kumar 2014 [29]	–/India	M: 250/F: 181	> 35	Asian	35.6 ± 3.37	Non-random	Pearson	FBG: 166		Unadjusted	Patients of a clinic	9/10
Aoi 2014 [22]	–/Japan	F: 64	62.4 ± 7.1	Asian	32.0 ± 1.6	Non-random	Pearson	HOMA-IR:0.263 HbA1c:0.298		Age	Healthy	9/10
Yan 2014 [30]	–/China	M = 971	Over 65	Asian	37.8 ± 2.8	Random	Pearson	FBG: 0.2		Unadjusted	Healthy	8/10
		F: 1121	years		34.4 ± 2.7			FBG: 0.2				
Torriani 2014 [27]	–/USA	M = 152	55 ± 17	NR	44 ± 6	Consecutive	Pearson	FBG:0.28		Age, disease status, sex	Healthy	10/10
		F = 151			39 ± 7			FBG: NS				
Wang 2015 [14]	–/China	M: 1144	20–65	Asian	39.4 ± 6.92	Random	Pearson	FBG: 0.25 HOMA-IR: 0.18		Unadjusted	Healthy	8/10
		F: 2163			36.2 ± 4.31			FBG: 0.06 HOMA-IR: 0.29				
Liang 2015 [11]	CRC/China	M: 1008	18–93	Asian	37.7 ± 2.49	Random	Pearson	FBG: 0.054		Age	Healthy	9/10
		F: 701			32.7 ± 2.30			FBG: 0.161				
Li 2015 [31]	SPECT/China	M: 744	50.1 ± 14.09 (18–89)	Asian	34.5 ± 2.15	Random	Pearson	FBG: 0.06 Insulin: 0.11 HOMA-IR: 0.12 HbA1c: 0.11		Age	Healthy	9/10
		F: 1924			31.0 ± 2.22			FBG: 0.10 Insulin: 0.09 HOMA-IR: 0.17 HbA1c: 0.06				
Baena 2016 [15]	ELSA/Brazil	M: 3810	62.4 ± 7.1 (35–74)	Spanish	38.9 ± 2.6	Random	Pearson	FBG: 0.193 Insulin: 0.415 HOMA-IR: 0.443		Age	Healthy	9/10
		F: 4916			33.0 ± 2.6			FBG: 0.218 Insulin: 0.337 HOMA-IR: 0.400				
Joshipura 2016 [12]	SOALS/USA	M: 329/F: 877	45–65	NR	(42.0 ± 4.8) (36.1 ± 2.9)	Random	Pearson	FBG: 0.10 HOMA-IR: 0.45 HbA1c: 0.28		Age, gender, smoking status & physical activity	Overweight or obese	10/10
Selvan 2016 [32]	–/India	M: 258	30–80	Asian	35.5 ± 17.0	Non-random	Pearson	FBG: 0.025 HbA1c: 0.024		Age	Healthy	9/10
		F: 193			32.0 ± 19.0							
								FBG: 0.221 HbA1c: 0.144				
Assyov 2017 [35]	–/Bulgaria	M: 102	49 ± 12 (45–70)	White	41.0 ± 4.0	Non-random	Pearson	FBG: 0.338 Insulin: 0.465 HOMA-IR: 0.385 HbA1c: 0.215		Age	Healthy	9/10
		F: 153			38.0 ± 3.0			FBG: 0.485 Insulin: 0.318 HOMA-IR: 0.369 HbA1c: 0.183				
Jiang 2017 [33]	–/China	M: 3369	≥ 40 60.0 ± 7.8	Asian	38.2 ± 2.63	Random	Pearson	FBG: 0.11 HbA1c: 0.01		Unadjusted	Healthy	8/10
		F: 5062			33.9 ± 2.45			FBG: 0.19 HbA1c: 0.03				
Zhong 2017 [9]	–/China	M: 965	≥ 65 37.21 ± 6.72	Asian	37.8 ± 2.80	Consecutive	Spearman	FBG: 0.195		Unadjusted	Elders/healthy	8/10
		F: 1109			34.4 ± 2.75			FBG: 0.194				

*NC* neck circumference, *WC* waist circumference, *HC* hip circumference, *NR* not reported, *M* male, *F* female, *FBG* fasting blood glucose, *HOMA-IR* homeostatic model assessment of insulin resistance, *HbA1c* hemoglobin A1c, *SD* standard deviation

^a^Based on Newcastle–Ottawa quality assessment scale (adapted for cross sectional studies) [23]

Four outcomes including FBG, serum fasting insulin level, HOMA-IR and HbA1c were examined in the eligible studies. The association between NC, FBG, serum fasting insulin level, HOMA-IR, and HbA1c were evaluated in 20, 8, 9 and 9 studies, respectively. The range of correlation coefficient for FBG was between 0.025 and 0.48; for serum fasting insulin level was between 0.09 and 0.50; for HOMA-IR was between 0.12 and 0.51, and for HbA1c was from 0.01 to 0.3. The associations between NC and glycemic parameters in 18 studies were evaluated by Pearson correlation and in 3 other studies by Spearman correlation coefficient.

### Meta-analysis of the correlation coefficient between NC and FBG

**Fig. 2:**
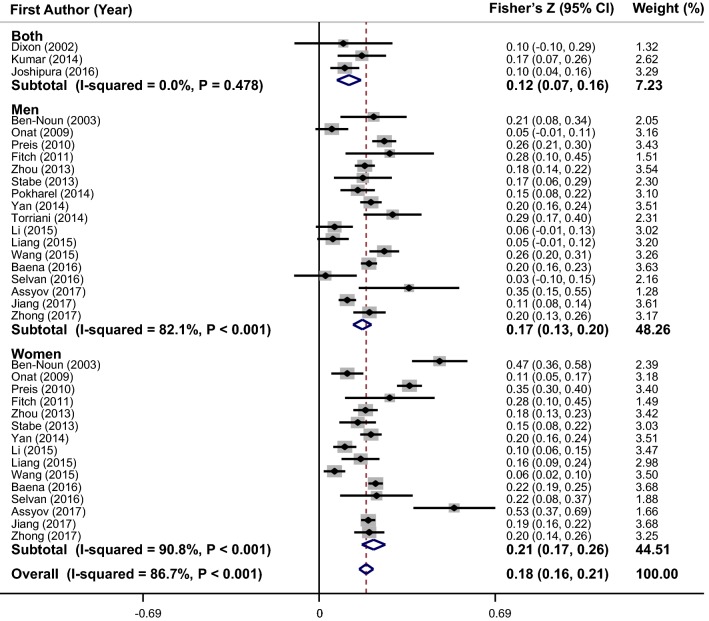
Forest plots of the correlation between neck circumference and fasting blood sugar (FBG)

**Fig. 3:**
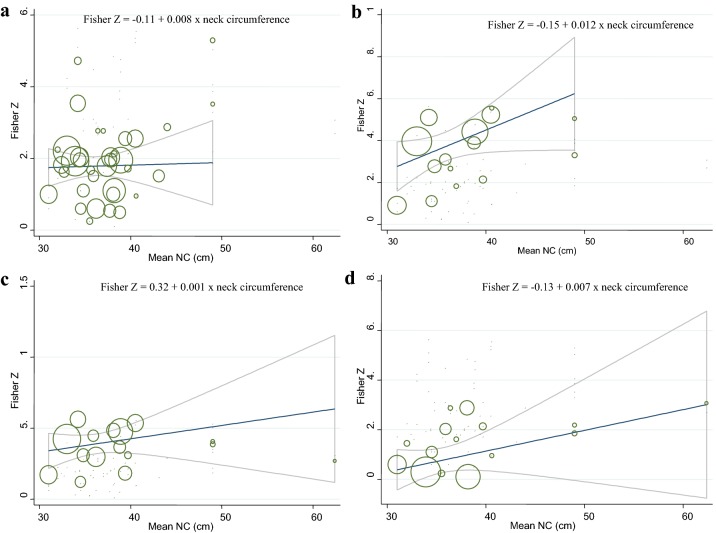
**a** Association between mean neck circumference values and glycemic profiles: meta-regression analysis. The means of neck circumference (cm) were modeled using a linear trend with random-effects meta-regression models. The solid line represents the weighted regression line based on variance-weighted least squares. The gray lines show the 95% CI around the regression line. The circles indicate Fisher Z in each study. The circle size is proportional to the precision of the Fisher Z. For fasting blood sugar: β = 0.008, P = 0.09, I^2^ residual = 87.08%. **b** For serum fasting insulin level: β = 0.012, P = 0.12, I^2^ residual = 94.63%. **c** For homeostasis model assessment-estimated insulin resistance: β = 0.001, P = 0.83, I^2^ residual = 95.74%. **d** For glycated hemoglobin: β = 0.007, P = 0.11, I^2^ residual = 87.07%

Thirty-five effect sizes on correlation between NC and FBG were derived from 20 studies (Fig. 2). Pooled results from included studies showed a positive correlation between NC and FBG (overall Fisher’s Z = 0.18; 95% CI 0.16–0.21). Heterogeneity was significant among included studies (I^2^ = 86.7%, P < 0.001). To find the source of heterogeneity, the subgroup analysis based on gender (Fig. 2), race, adjustments, correlation type, health status, and sampling method was conducted (Table 2). Heterogeneity was not completely eliminated in all subgroups; however, there was no heterogeneity between studies in the several subgroups. As shown in Fig. 3a, meta-regression of the studies indicated that NC (as a continuous variable) was marginally associated with FBG values in a dose–response manner (β = 0.008, P = 0.09). Sensitivity analysis showed that the overall estimate was not changed after recalculation of the overall effect size by sequentially elimination of each study at a time. There was no evidence of substantial publication bias (Begg’s test = 0.51 and Egger’s test = 0.63).

**Table 2:** Results of subgroup-analysis for neck circumference and glycemic parameters

	No. of effect sizes	Fisher’s Z (95% CI)	P within^a^	I^2^ (%)	P between^b^
Subgroup analyses for NC and FBG					
Race					< 0.001
USA	8	0.23 (0.15 to 0.31)	< 0.001	87.1	
Mideast	4	0.21 (0.04 to 0.37)	< 0.001	93.4	
Asian	17	0.15 (0.12 to 0.18)	< 0.001	82.0	
Latin America	4	0.20 (0.17 to 0.23)	0.241	28.5	
European	2	0.45 (0.28 to 0.62)	0.170	46.9	
Adjustments					0.055
Yes	22	0.18 (0.14 to 0.21)	< 0.001	87.5	
No	13	0.20 (0.15 to 0.24)	< 0.001	85.8	
Correlation type					0.059
Pearson	30	0.18 (0.15 to 0.21)	< 0.001	87.5	
Spearman	5	0.25 (0.14 to 0.36)	< 0.001	82.1	
Health status					0.045
Patients	4	0.17 (0.07 to 0.28)	0.084	54.8	
Healthy	31	0.19 (0.16 to 0.21)	< 0.001	87.7	
Sampling method					0.007
Consecutive	6	0.25 (0.16 to 0.34)	< 0.001	79.5	
Random	19	0.16 (0.13 to 0.20)	< 0.001	90.5	
Non-random	10	0.21 (0.15 to 0.28)	< 0.001	71.4	
Subgroup analyses for NC and serum fasting insulin level					
Race					< 0.001
USA	5	0.43 (0.34 to 0.53)	< 0.001	81.1	
Mideast	2	0.33 (0.22 to 0.44)	0.015	83.1	
Asian	2	0.10 (0.06 to 0.13)	0.640	0.0	
Latin America	4	0.36 (0.29 to 0.43)	< 0.001	87.0	
European	2	0.41 (0.24 to 0.58)	0.178	44.8	
Adjustments					0.35
Yes	12	0.34 (0.25 to 0.42)	< 0.001	96.5	
No	3	0.33 (0.12 to 0.55)	0.014	76.5	
Correlation type					0.016
Pearson	11	0.34 (0.25 to 0.43)	< 0.001	96.8	
Spearman	4	0.32 (0.22 to 0.41)	0.089	54.0	
Health status					0.35
Patients	3	0.32 (0.22 to 0.42)	0.014	76.5	
Healthy	12	0.37 (0.36 to 0.38)	< 0.001	96.5	
Sampling method					0.001
Consecutive	1	0.55 (0.36 to 0.75)	–	–	
Random	8	0.34 (0.24 to 0.45)	< 0.001	97.7	
Non-random	6	0.29 (0.22 to 0.36)	0.137	40.2	
Subgroup analyses for NC and HOMA-IR					
Race					< 0.001
USA	3	0.53 (0.49 to 0.57)	0.122	52.6	
Mideast	2	0.34 (0.28 to 0.39)	0.222	32.9	
Asian	5	0.20 (0.13 to 0.270	< 0.001	85.0	
Latin America	4	0.43 (0.39 to 0.48)	0.010	73.4	
European	2	0.39 (0.27 to 0.52)	0.885	0.0	
Adjustments					< 0.001
Yes	14	0.38 (0.31 to 0.45)	< 0.001	95.3	
No	2	0.24 (0.13 to 0.36)	0.001	90.1	
Correlation type					0.547
Pearson	12	0.35 (0.27 to 0.43)	< 0.001	96.8	
Spearman	4	0.40 (0.33 to 0.47)	0.248	27.3	
Health status					0.001
Patients	1	0.48 (0.43 to 0.54)	–	–	
Healthy	15	0.35 (0.28 to 0.42)	< 0.001	95.9	
Sampling method					0.701
Random	11	0.36 (0.28 to 0.44)	< 0.001	97.1	
Non-random	5	0.39 (0.32 to 0.46)	0.266	23.3	
Subgroup analyses for NC and HbA1c					
Race					< 0.001
USA	4	0.23 (0.15 to 0.32)	0.168	40.6	
Asian	7	0.05 (0.02 to 0.09)	0.025	58.4	
Latin America	2	0.21 (0.15 to 0.27)	0.879	0.0	
European	2	0.20 (0.07 to 0.32)	0.797	0.0	
Adjustments					< 0.001
Yes	10	0.17 (0.10 to 0.24)	< 0.001	81.6	
No	5	0.06 (0.00 to 0.11)	0.015	67.4	
Correlation type					< 0.001
Pearson	11	0.12 (0.06 to 0.18)	< 0.001	88.9	
Spearman	4	0.20 (0.15 to 0.26)	0.992	0.0	
Health status					< 0.001
Patients	4	0.23 (0.15 to 0.32)	0.168	40.6	
Healthy	11	0.11 (0.06 to 0.15)	< 0.001	78.7	
Sampling method					< 0.001
Consecutive	1	0.10 (− 0.10 to 0.29)	–	–	
Random	5	0.10 (0.01 to 0.18)	< 0.001	94.8	
Non-random	9	0.18 (0.13 to 0.23)	0.281	18.2	

^a^P for heterogeneity, within subgroup

^b^P for heterogeneity, between subgroup

### Meta-analysis of the correlation coefficient between NC and serum fasting insulin levels

**Fig. 4:**
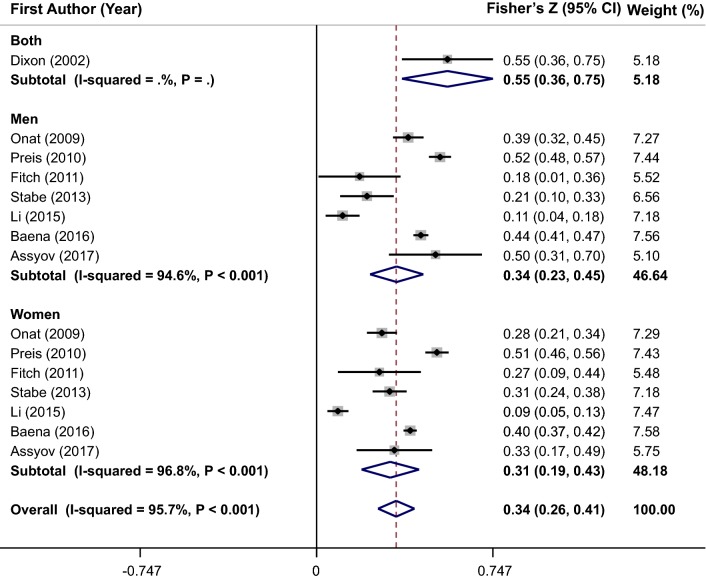
Forest plots of the correlation between neck circumference and serum fasting insulin level

A combination of 15 effect sizes from 8 studies revealed that NC was positively correlated with serum fasting insulin level (overall Fisher’s Z = 0.34; 95% CI 0.26–0.41) (Fig. 4). Because of the significant heterogeneity between studies (I^2^ = 95.7%, P < 0.001), we performed subgroup analysis based on gender (Fig. 4), race, adjustments, correlation type, health status, and sampling method to investigate its sources (Table 2). Although subgroup analysis could not detect potential sources of observed heterogeneity, the between-studies heterogeneity was eliminated in Asian and European sub-groups and non-random groups. Meta-regression analysis showed that increment in NC values was not significantly associated with serum fasting insulin level in a linear manner (β = 0.012, P = 0.12) (Fig. 3b). Sensitivity analysis revealed that the pooled estimate did not substantially change with the omission of the studies one at a time. No significant evidence of publication bias was found by Begg’s test (P = 0.73) and Egger’s test (P = 0.44).

### Meta-analysis of the correlation coefficient between NC and HOMA-IR

**Fig. 5:**
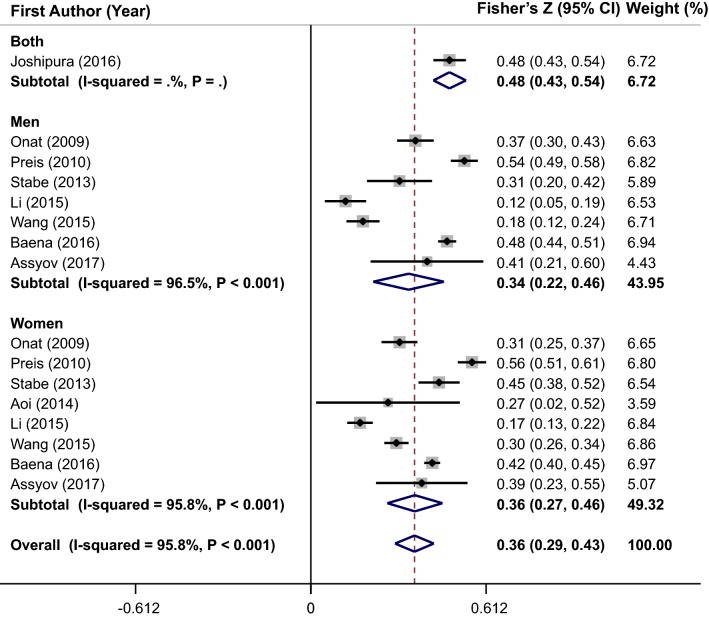
Forest plots of the correlation between neck circumference and homeostasis model assessment-estimated insulin resistance (HOMA-IR)

Pooling 16 effect sizes from 9 studies, NC was significantly correlated with HOMA-IR (overall Fisher’s Z = 0.36; 95% CI 0.29–0.43) (Fig. 5). A significant heterogeneity was evident between studies (I^2^ = 95.8%, P < 0.001). The heterogeneity was not completely eliminated by subgroup analysis according to gender (Fig. 5), race, adjustments, correlation type, health status, and sampling method (Table 2). Sub-group analysis by race showed that the heterogeneity was eliminated in all sub-groups except Latin America and Asian populations. In addition, heterogeneity was not observed in non-random and spearman sub-groups. The results of meta-regression illustrated that there was a non-significant linear trend between NC measurements (cm) and HOMA-IR (β = 0.001, P = 0.83) (Fig. 3c). The sensitivity analysis was performed and the pooled estimate did not significantly change after exclusion of each study at a time. Results of Egger’s test (P = 0.382) and Begg’s test (P = 0.528) indicated no evidence of publication bias.

### Meta-analysis of the correlation coefficient between NC and HbA1c

**Fig. 6:**
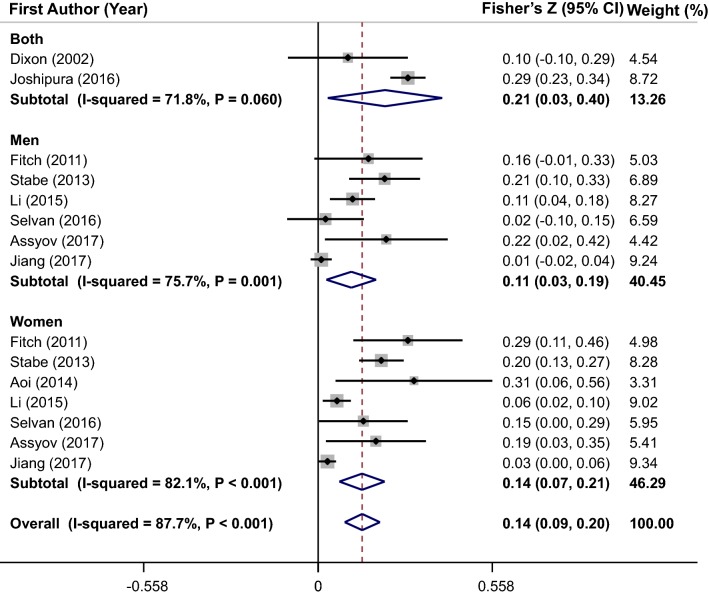
Forest plots of the correlation between neck circumference and glycated hemoglobin (HbA1c)

The significant positive correlation between NC and HbA1c was suggested by pooled estimate of 15 effect sizes from 9 studies (overall Fisher’s Z = 0.14; 95% CI 0.09–0.20) (Fig. 6). Between-studies heterogeneity was significant (I^2^ = 87.7%, P < 0.001); thus, the subgroup analysis based on gender (Fig. 6), and other confounders was performed (Table 2). The heterogeneity was not completely eliminated by these stratified analyses, although the heterogeneity was removed in some sub-groups. Meta-regression analysis of studies showed that there was no significant linear association between NC values (cm) and HbA1c (β = 0.007, P = 0.11) (Fig. 3d). Results of sensitivity analysis revealed that overall estimate was not affected by elimination of each study. In addition, no evidence of publication bias was seen (P = 0.22 by Begg’s test).

## Discussion

This meta-analysis of observational studies showed that NC was positively correlated with FBG, serum fasting insulin level, HOMA-IR, and HbA1c. The findings were not varied by gender, race, adjustments, correlation type, health status, and sampling method. Furthermore, meta-regression analysis showed that NC were marginally associated with FBG in a linear manner. These findings suggested that NC, as a simple and appropriate tool, could be used in clinical screening of glycemic parameters and prediction of type 2 diabetes. To our knowledge, the current study was comprehensively reviewed the correlation between NC and glycemic parameters for the first time.

Recently, NC was considered as a useful tool for measurement of overweight and obesity. Hingorjo et al. reported that NC is good predictor of overweight and obesity and suggested that the cut-off point of NC for overweight and obesity in male and female is ≥ 35.5 cm and ≥ 32 cm, respectively [36]. In addition, other studies reported that there are significant positive correlations between NC and weight, BMI, waist circumference, waist to hip ratio as well as metabolic syndrome in different populations [37, 38].

Several studies suggested that NC might have a role in prevalence of chronic disease including cardiovascular diseases, metabolic syndrome, and diabetes. Increasing in NC might result in dyslipidemia and elevated risk of cardiovascular diseases [39]. Also, a number of studies reported that large NC values might increase risk of inflammation and cardio-vascular disease [40, 41]. A recent meta-analysis found no significant association between NC and metabolic syndrome; however, the mentioned study reported significant positive associations between NC and the components of metabolic syndrome [16]. Several eligible studies [11, 12, 17] have been missed by the search in this investigation; so, the results might be distorted by defective search strategy. Another meta-analysis in 2018 assessed the relationship between NC and cardio-metabolic risk factors and reported positive and significant correlations between NC and with two glycemic indices (FBS and HOMA-IR). This analysis was included only 4 studies for FBS and 3 investigations for HOMA-IR in adult population [42]. Several eligible studies were missed in the mentioned meta-analysis and the results might be distorted by defective search strategy. We tried to consider all published data in this field and provided more accurate information in the present study.

The association between NC and glucose intolerance was evaluated in some previous studies. Laakso et al. study reported that the risk of glucose intolerance and hyperinsulinemia was higher in the highest quintile of NC compared with the lowest one [43]. Another study compared the correlation of different anthropometric measurements including BMI, waist, hip and neck circumferences with visceral adiposity and HOMA-IR. This study suggested that NC outstripped other anthropometric measurements in prediction of insulin resistance as well as visceral adipose tissue [44]. These findings were in line with current meta-analysis. In addition, one study assessed the NC and other anthropometric measurements in diabetic and non-diabetic subjects [45]. Although mean NC in diabetic patients was higher than non-diabetic individuals in this study, the cut-off point of NC in diabetic (> 36 cm) was less than non-diabetic subjects (> 37 cm). This finding revealed that a broad assessment of obesity is needed in diabetic subjects [45].

NC was considered as an estimation of upper-body subcutaneous adipose tissue that might have a role in prediction of insulin resistance and type-2 diabetes [25, 34, 46]. Excess systematic free fatty acid might be one mechanism to explain the correlation between NC and insulin resistance. The concentration of free fatty acids (FFAs) is affected by NC values. In other words, the lipolytic function and FFAs release rate of upper-body subcutaneous fat is more than lower-body subcutaneous fat [47, 48]. The elevated systemic FFAs had a role in increased very low density lipoprotein production and inhibition of insulin clearance that lead to insulin resistance [49–51]. Also, NC was positively correlated with whole body and visceral fat that both were associated with biological parameters of insulin resistance [5, 36, 39]. Two perivascular ectopic fat depots were also found in neck region. Secretion of adipokines, such as leptin, adiponectin, and interleukin 6 from these fat depots might result in metabolic dysfunction including insulin resistance [22, 45, 52, 53]. In addition, subjects with large NC had more risk for obesity [37, 38]. Insulin resistance is one of the important complication of obesity that has an ability to engender hyperglycemia and impaired glycemic parameters [54, 55].

Current meta-analysis has some strengths and limitations. First, contribution of a large number of subjects increases the statistical power. Publication bias was not observed in the analysis and the comprehensive subgroup analyses were conducted based on different potential confounders such as gender, race, adjustments, correlation type, health status, and sampling method. Several limitations of the current study merit discussion. All of the included studies in the meta-analysis had observational design, thus we could not infer a causal association between NC and glycemic parameters. In addition, most of included studies did not make adjustment for the potential confounders, especially dietary intakes. So, the residual confounder might influence the correlation between NC and glycemic parameters. In addition, the direct and quantify measure of depot of fat might not be explained by single measurement of NC, because measure of NC involved both adipose and lean tissue. Finally, the heterogeneity between studies was not completely eliminated after subgroup analysis and meta-regression.

## Conclusions

In conclusion, this meta-analysis of observational studies showed that neck circumference was positively correlated with glycemic parameters including FBG, serum fasting insulin level, HOMA-IR, and HbA1c. However, further studies with prospective design are required to confirm these findings.

## Data Availability

Data are available on request.

## Abbreviations

NC: neck circumference
FBG: fasting blood glucose
FFA: free fatty acids
HOMA-IR: homeostasis model assessment-estimated insulin resistance
HbA1c: glycated hemoglobin
